# Dysfunction of the magnocellular subdivision of the visual thalamus in developmental dyslexia

**DOI:** 10.1093/brain/awae235

**Published:** 2024-08-07

**Authors:** Christa Müller-Axt, Louise Kauffmann, Cornelius Eichner, Katharina von Kriegstein

**Affiliations:** Faculty of Psychology, TUD Dresden University of Technology, 01062 Dresden, Germany; Neural Mechanisms of Human Communication, Max Planck Institute for Human Cognitive and Brain Sciences, 04103 Leipzig, Germany; Neural Mechanisms of Human Communication, Max Planck Institute for Human Cognitive and Brain Sciences, 04103 Leipzig, Germany; Laboratory of Psychology and NeuroCognition, Grenoble Alpes University, 38000 Grenoble, France; Department of Neuropsychology, Max Planck Institute for Human Cognitive and Brain Sciences, 04103 Leipzig, Germany; Faculty of Psychology, TUD Dresden University of Technology, 01062 Dresden, Germany

**Keywords:** reading disability, lateral geniculate nucleus, visual processing, predictive coding, fMRI, quantitative MRI

## Abstract

Developmental dyslexia (DD) is one of the most common learning disorders, affecting millions of children and adults worldwide. To date, scientific research has attempted to explain DD primarily based on pathophysiological alterations in the cerebral cortex. In contrast, several decades ago, pioneering research on five post-mortem human brains suggested that a core characteristic of DD might be morphological alterations in a specific subdivision of the visual thalamus—the magnocellular lateral geniculate nucleus (M-LGN). However, due to considerable technical challenges in investigating LGN subdivisions non-invasively in humans, this finding was never confirmed *in vivo*, and its relevance for DD pathology remained highly controversial.

Here, we leveraged recent advances in high resolution MRI at high field strength (7 T) to investigate the M-LGN in DD *in vivo*. Using a case-control design, we acquired data from a large sample of young adults with DD (*n* = 26; age 28 ± 7 years; 13 females) and matched control participants (*n* = 28; age 27 ± 6 years; 15 females). Each participant completed a comprehensive diagnostic behavioural test battery and participated in two MRI sessions, including three functional MRI experiments and one structural MRI acquisition. We measured blood oxygen level-dependent responses and longitudinal relaxation rates to compare both groups on LGN subdivision function and myelination. Based on previous research, we hypothesized that the M-LGN is altered in DD and that these alterations are associated with a key DD diagnostic score, i.e. rapid letter and number naming.

The results showed aberrant responses of the M-LGN in DD compared to controls, which was reflected in a different functional lateralization of this subdivision between groups. These alterations were associated with rapid letter and number naming performance, specifically in male DD. We also found lateralization differences in the longitudinal relaxation rates of the M-LGN in DD relative to controls. Conversely, the other main subdivision of the LGN, the parvocellular LGN (P-LGN), showed comparable blood oxygen level-dependent responses and longitudinal relaxation rates between groups.

The present study is the first to unequivocally show that M-LGN alterations are a hallmark of DD, affecting both the function and microstructure of this subdivision. It further provides a first functional interpretation of M-LGN alterations and a basis for a better understanding of sex-specific differences in DD with implications for prospective diagnostic and treatment strategies.

## Teaser

Prior post-mortem research has linked developmental dyslexia to histological alterations in the magnocellular lateral geniculate nucleus (M-LGN), but this finding has lacked confirmation *in vivo*. Using 7 T MRI, Müller-Axt *et al.* reveal extensive M-LGN alterations in people with dyslexia and link them to a key symptom in male subjects.

## Introduction

Developmental dyslexia (DD) is a neurodevelopmental disorder characterized by persistent difficulties in acquiring effective literacy skills despite adequate intellectual development, motivation and educational opportunities.^1^ With a 5%–10% prevalence in children, DD encompasses the most common learning disorder. DD is often associated with considerable long-term consequences for the individual and high costs for society.^1,2^ Compared to typically reading peers, DD is associated with significantly higher academic drop out and unemployment rates, poorer health and a shortened life expectancy.^1^

Research on the neurobiological origins of DD in humans focuses primarily on the cerebral cortex and has revealed alterations particularly in a left-lateralized language network.^3^ However, this cortico-centric view of DD is challenged by histopathological observations made in the early 1990s on several post-mortem brains of dyslexics.^4,5^ These studies revealed that DD is not only associated with alterations (neuronal ectopias and focal microgyria) in key cortical language regions but also with histological alterations of the sensory thalami, i.e. the lateral geniculate nucleus (LGN) and the medial geniculate body (MGB) of the visual and auditory processing pathway, respectively.^4,5^ Sensory thalami are the last subcortical processing site before sensory information is routed to primary cortices.^6^ Sensory thalamus alterations, including sex-specific differences, have also been observed in several animal models of DD.^7,8^*In vivo* MRI studies in humans have shown that thalamo-cortical connectivity in DD is altered predominantly in the left hemisphere in the visual pathway, mirroring similar findings in the auditory pathway.^9,10^

In humans, the LGN is a small, layered structure that can be coarsely partitioned into two main subdivisions: a magnocellular (M-LGN; layers 1–2) and a parvocellular (P-LGN; layers 3–6) subdivision.^11,12^ Thin intercalated koniocellular layers separate each of the individual M and P layers. Neurons of the M and P subdivisions process complementary visual information, e.g. M-LGN neurons are involved in coarse spatial image analysis and are specialized in detecting rapid visual changes and motion. Conversely, P-LGN neurons are involved in processing colour and fine spatial detail. The functional role of koniocellular neurons is still largely unresolved; however, they also contribute to colour vision.^6^ Human post-mortem studies in DD demonstrated morphological alterations specifically in the magnocellular layers but not in the parvocellular layers of the LGN.^4^ These findings were based on *n* = 5 post-mortem dyslexic cases and have not yet been replicated in further human post-mortem or *in vivo* imaging studies. Previous *in vivo* studies investigating LGN alterations in DD have provided unreliable results.^13^ Furthermore, they have been limited to measuring the entire LGN and therefore lack the sensitivity to assess individual M and P subdivisions and their potential role in DD.^14,15^ In addition, measuring magnocellular function behaviourally is indirect and unreliable^16^ and not all dyslexics exhibit behavioural impairments that could be attributed to a general magnocellular visual processing difficulty.^17^ Thus, to date, it remains elusive (i) whether M-LGN alterations can also be detected in DD *in vivo*; and if so (ii) what functional relevance these may have for dyslexia symptoms.

The scarcity of clinical post-mortem brain specimens and the technical challenges associated with *in vivo* MRI measurements of small subcortical brain structures pose major obstacles to answering these questions. In humans, individual LGN layers are ≤1 mm thick, verging on the limits of attainable image resolutions of conventional MRI.^11,12^ However, recent advances in high-field MRI have made it possible to measure distinct signals from the M- and P-LGN in humans *in vivo*, paving the way for assessing subdivision-specific LGN alterations in larger sample sizes.^12,18^

**Figure 1:**
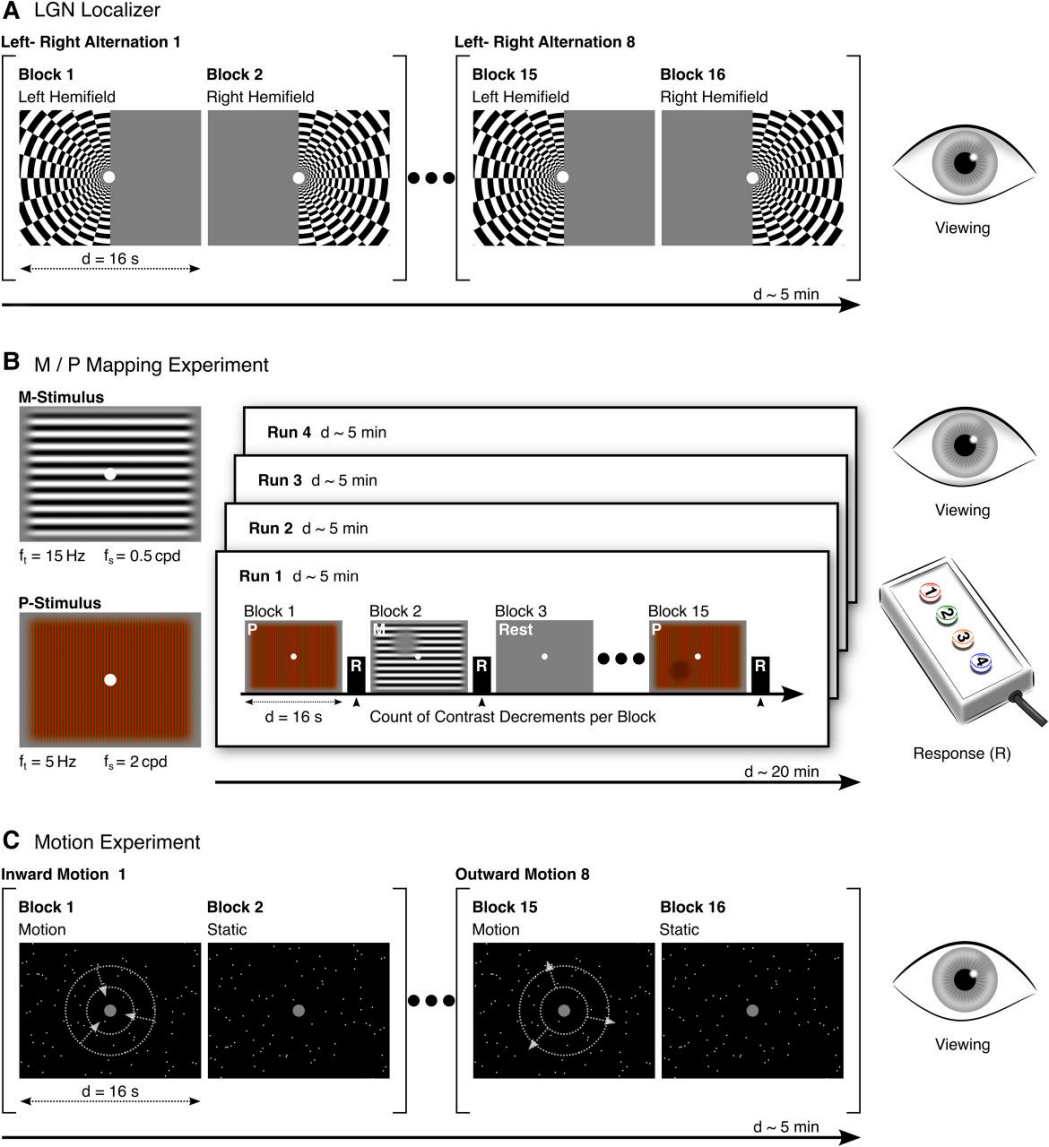
**Experimental design of the three fMRI experiments.** (**A**) In the lateral geniculate nucleus (LGN) localizer experiment, participants saw a flickering checkerboard stimulus in blocks alternating between the left and right visual hemifields. Participants viewed the stimuli while maintaining central fixation. (**B**) During the magnocellular/ parvocellular (M/P) mapping experiment, participants viewed two types of experimental stimulus blocks designed to evoke different BOLD responses from the M- and P-LGN. M-blocks consisted of a full-field achromatic grating stimulus with a luminance contrast of 100%, presented at a low spatial (f_s_) and a high temporal frequency (f_t_). P-blocks consisted of a full-field coloured grating stimulus with low luminance contrast, presented at a higher spatial and a lower temporal frequency. M- and P-blocks were interleaved with rest blocks containing a grey screen. During the experimental stimulus blocks, participants detected contrast decrements (luminance in M-blocks, colour in P-blocks) and reported the number of targets by button press after each block (R). (**C**) In the motion experiment, participants were presented with blocks of either moving or static point clouds. Motion blocks contained either inward or outward motion. Participants viewed the stimuli while maintaining central fixation. Refer to the ‘Materials and methods’ section for more details on the experimental designs. d = duration; cpd = cycles per degree.

We used recently developed high-field functional MRI (fMRI) experiments at 7 T to investigate whether DD is associated with functional alterations of the M-LGN. In addition, we acquired quantitative MRI data to explore potential structural alterations of the M-LGN. We acquired data from a large sample of *n* = 54 young German adults with a lifelong history of DD and matched control participants (Supplementary Table 1). With this sample we performed three blood oxygen level-dependent (BOLD) fMRI experiments (Fig. 1). The central aim of these experiments was to test whether (i) M-LGN alterations can also be detected in DD *in vivo*; and if so (ii) whether they are related to a dyslexia diagnostic score, i.e. rapid automatized naming of letters and numbers (RANln). We focused specifically on RANln performance because it is associated with altered connectivity between the LGN and the cerebral cortex in DD^10^ and with sensory thalamus dysfunction in the auditory modality.^19^ Furthermore, RANln performance is key for predicting reading ability.^20^

## Materials and methods

### Participants

Fifty-four healthy adult German speakers were included in the study. This sample consisted of 26 participants with DD and 28 control participants, matched in age, sex, handedness and non-verbal IQ (Supplementary material, ‘Methods’ section and Supplementary Table 1). Participants with DD performed worse than controls on tests of literacy (spelling, reading speed and comprehension), rapid automatized naming of letters and numbers (RANln), and word and non-word reading (Supplementary material, ‘Methods’ section and Supplementary Table 1).

### MRI experiments

#### Procedure

Participants attended two MRI sessions on two separate days. The sessions included three fMRI experiments: the LGN localizer and M/P mapping experiment (first session) and a motion experiment (second session). In addition, whole-brain quantitative structural magnetic resonance images were acquired in each participant during the first session. One DD participant attended only the first MRI session due to pregnancy at the time of the second session. In the context of a different research question, we acquired additional fMRI and diffusion-weighted imaging data from the participants, the results of which will be reported elsewhere.

#### Set-up

In each fMRI experiment, visual stimuli were front-projected onto a translucent screen positioned on the participants’ chests. Participants viewed the screen in the MRI system through a mirror mounted directly above their eyes. During the fMRI experiments, we also recorded cardio-respiratory data from the participants. This was done to account for physiological noise in the BOLD signal during data processing to increase the signal-to-noise ratio in the LGN.^21^ For more details on display settings, visual stimulation software and physiological recordings, refer to the Supplementary material, ‘Methods’ section.

### Experimental design

#### LGN localizer

This experiment was used to functionally localize the LGN in each participant (Fig. 1A).^18^ The stimulus consisted of a flickering radial checkerboard with 100% contrast, with its contrast polarity reversed at 4 Hz (for the full cycle). The checkerboard covered half the screen while the other half contained a uniform grey background. The checkerboard alternated between the two visual hemifields in a block design. Participants maintained fixation on a central white fixation dot while viewing the stimuli. Each hemifield block lasted 16 s, and the whole run was composed of eight left-right alternations for a total of 16 blocks and a run duration of 5 min. Further details on the experimental design can be found in Denison *et al*.^18^

#### M/P mapping

This experiment used full-field stimuli designed to match the selective response properties of neurons in the M-LGN and P-LGN (Fig. 1B).^18^ The M-stimulus was a sinusoidal greyscale grating with a luminance contrast of 100%, a low spatial frequency of 0.5 cycles per degree (cpd) and a sinusoidal counterphase flicker frequency of 15 Hz. The P-stimulus was a sinusoidal high colour-contrast red-green grating with low luminance contrast, a higher spatial frequency of 2 cpd and a lower sinusoidal counterphase flicker of 5 Hz. Gratings changed orientation every 3 s and could be presented at one of six orientations (0°, 30°, 60°, 90°, 120°, 150°). M- and P-stimuli were presented in a block design and were interspersed with rest blocks consisting of a uniform grey background. Throughout the experiment, participants maintained fixation on a central white fixation dot while viewing the stimuli. To ensure continued fixation on the screen during experimental M/P-blocks, participants were asked to detect contrast decrements (0–3 targets) that could appear at random locations within each block. At the end of each block, participants had 1.5 s to report the number of targets per button press. Each block lasted 16 s and each run was composed of six M-blocks, six P-blocks and three rest blocks for a total of 15 blocks. Participants completed four runs of the M/P mapping experiment, which lasted ∼5 min each. Further details on the experimental design can be found in Denison *et al*.^18^

#### Motion experiment

This experiment served to functionally validate the obtained M- and P-subdivision maps and consisted of alternating moving and static point clouds presented in a block design (Fig. 1C).^22^ In the motion blocks, point clouds consisted of 250 white dots with a radius of 0.1°, moving radially against a black background at a speed of 4.7 °/s and 100% coherence within a circular aperture of 17°. For half of the motion blocks, the points moved inward, while for the other half, they moved outward. Radial motion was chosen to facilitate central fixation and to stimulate a broad spectrum of motion direction-selective cells.^23^ During static blocks, the same number of dots was displayed at random locations and remained stationary for the duration of the block. Throughout all blocks, participants were instructed to maintain fixation on a central grey fixation point (0.2° radius) while viewing the stimuli. Each block lasted 16 s, and a run was composed of eight blocks of each type (i.e. motion and static), for a total of 16 blocks. Participants completed one run, which lasted ∼5 min.

### High resolution MRI data acquisition

High resolution functional and structural MRI data were acquired on a 7 T Magnetom MRI system (Siemens Healthineers) equipped with a 32-channel head coil (Nova Medical). In the three fMRI experiments, high resolution echo-planar images were acquired at a resolution of 1.25 × 1.25 × 1.2 mm and partial brain coverage (40 transverse slices), covering the LGN and visual cortex. High resolution whole-brain quantitative structural magnetic resonance images were acquired (0.7 mm isotropic resolution) for registration purposes and an exploratory analysis of LGN microstructure (Supplementary material, ‘Methods’ section). Participants received foam padding around the head to reduce head motion. For further details on the acquisition parameters and a quantitative evaluation of head motion, refer to the Supplementary material, ‘Methods’ section.

### MRI data processing

Preprocessing and first-level statistical analyses of fMRI data were performed using standard pipelines in SPM12 (Wellcome Centre for Human Neuroimaging, London, UK), implemented in MATLAB 2019Rb (MathWorks Inc., Natick, MA, USA) (Supplementary material, ‘Methods’ section).

### Definition of the LGN

We used a publicly available high resolution 7 T probabilistic LGN atlas to precisely segment the LGN in each participant and to carefully demarcate it from adjacent visual brain structures.^12^ Non-linear registrations of the atlas to each participant's native quantitative T_1_ image were performed using (landmark-based) symmetric normalization in ANTs (version 2.3.1; Supplementary material, ‘Methods’ section).^24^ For each participant, individual left and right LGN masks were then registered to the functional image data. We also verified whether the resulting masks overlapped with the functional responses obtained in the LGN localizer experiment.

### Definition of M- and P-LGN

M- and P-LGNs were defined using the M/P mapping experiment, as previously described.^18^ For each participant, we computed beta M-P maps in native space by subtracting the beta maps obtained from the general linear model (GLM) estimation corresponding to the M- and P-stimulus conditions of the M/P mapping experiment. It follows that voxels with larger values on the beta M-P maps indicate a higher response preference for the M-stimulus, while lower voxel values indicate a higher response preference for the P-stimulus. To confine these maps to relevant voxels within the LGN, individual beta M-P maps were then masked with the previously defined individual left and right LGN masks. For each participant and hemisphere separately, the M-LGN was defined as the 20% of voxels with the largest beta M-P values, while the remaining 80% of voxels formed the P-LGN. This 20/80% volume allocation criterion is based on previous histological studies showing that the proportion of M and P neurons in the human LGN falls within these bounds, respectively.^11^

**Figure 2:**
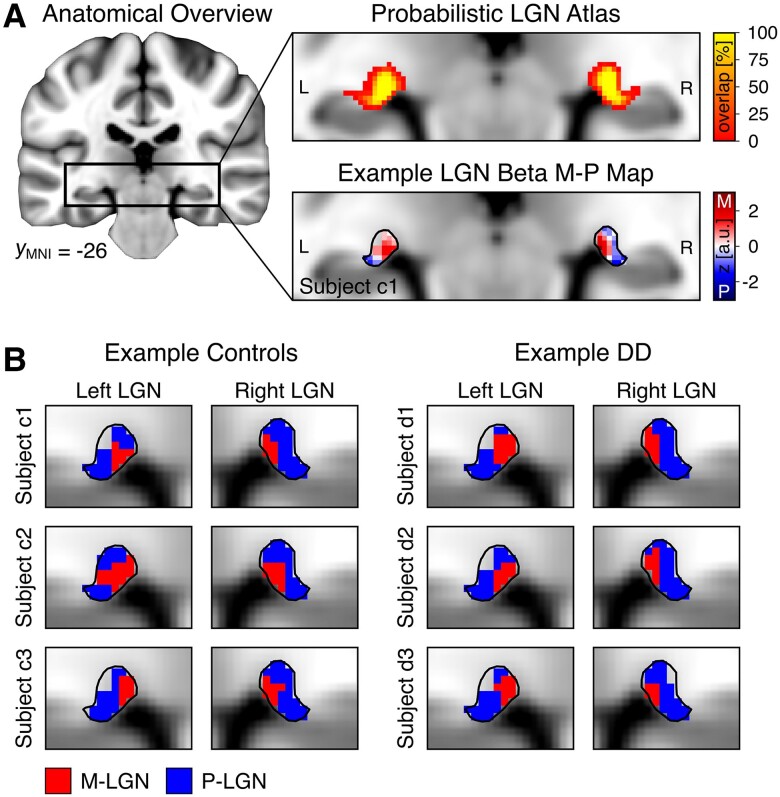
**Definition of M/P-LGN in control and developmental dyslexia participants.** (**A**) *Left*: Anatomical overview of the location of the lateral geniculate nucleus (LGN), indicated by the black rectangle, on the standard brain of the Montreal Neurological Institute (MNI). *Right*: We used a publicly available high resolution probabilistic LGN atlas (*top*) to confine functional responses from the LGN localizer to the bilateral nuclei in each participant.^12^ The atlas was set to a threshold of 35% overlap across subjects, as indicated by the black outlines surrounding the left and right LGN (*bottom*). M/P-LGN mapping was performed within these defined regions by applying a volume threshold of 20/80% to the individual beta M-P maps obtained from the M/P mapping experiment.^18^ An example beta M-P map is depicted in the *bottom* panel. On this map, LGN voxels with larger values (red colour) show a higher response preference for the M-stimulus, while voxels with lower values (blue colour) show a higher response preference for the P-stimulus. (**B**) Examples of derived M-LGN (in red colour) and P-LGN (in blue colour) subdivision maps based on the described volume threshold procedure in representative control and developmental dyslexia (DD) participants. a.u. = arbitrary units; M = magnocellular; P = parvocellular.

As a quality criterion, we checked whether the M/P-LGN subdivision maps defined in native space adhered to the anatomically known spatial configuration of the M-LGN being located more medially than the P-LGN.^12,18^ To this end, we computed individual M/P-LGN subdivision maps also in MNI standard space. This step permitted comparability between participants by aligning all LGNs in a common reference space. MNI beta M-P maps were masked with a probabilistic LGN atlas^12^ (in MNI 1 mm standard space) (Fig. 2A) and the same 20/80% volume allocation criterion was applied to define M- and P-LGN subdivision maps, respectively (Fig. 2B and Supplementary material, ‘Methods’ section). The analysis in MNI standard space served solely for quality control of the spatial configuration of the identified M/P-LGN subdivision maps. All further quantitative analyses comparing the accuracy of the M/P-LGN subdivision maps between groups are based on the data in participants’ native space.

### Extraction of signal change

Beta estimates corresponding to the conditions of interest were extracted from all voxels within the LGN (i.e. for the LGN localizer experiment) or the M- and P-LGN (i.e. for the M/P mapping and motion experiments) in each participant using an in-house toolbox. These estimates were then converted to per cent signal change (PSC). PSC was computed as: PSC = (β_condition_ × SF / β_constant_) × 100, wherein β_condition_ corresponds to the parameter estimate of the condition of interest, SF refers to the scale factor of the design matrix, and β_constant_ corresponds to the parameter estimate for the constant term.^25^ Mean PSC values were extracted from each region for every participant and experimental condition in the fMRI experiments, and subjected to mixed-design ANOVAs for statistical analysis Supplementary material, ‘Methods’ section). For the motion experiment, we used the identified M- and P-LGN subdivisions to mask responses in the contrast motion versus static.

### Statistical analyses

Mean PSC values were submitted to mixed-design ANOVAs for statistical analysis. Pairwise comparisons were performed using paired *t*-tests, where appropriate. Effect sizes for ANOVAs and *t*-tests were calculated using partial eta squared ($\eta_{p}^{2}$) and Cohen's *d*, respectively. Correlation analyses between PSC and RANln were performed using Pearson's correlations. Correlation analyses were only performed with RANln, while other behavioural measures were omitted. This choice was motivated by previous evidence showing an association between RANln and sensory thalamus alterations in DD.^10,19^ We checked the data for normality using the Shapiro-Wilk test.^26^ Participants whose results deviated more than two standard deviations (SD) from the group mean were excluded. This was the case for (i) one DD participant in the correlation analysis of M-LGN responses and RANln reaction times in female DD; (ii) three participants in the structural brain analysis of M-LGN R_1_ data (*n* = 1 control, *n* = 2 DD); and (iii) two participants (*n* = 1 control, *n* = 1 DD) in the P-LGN R_1_ data analysis. To account for deviations of the R_1_ data from a normal distribution despite outlier rejection, the R_1_ data of the M- and P-LGN were analysed using independent non-parametric Mann-Whitney *U*-tests. For all statistical tests, the significance level *α* was set to 0.05, and Bonferroni correction was applied as described later.

## Results

In each individual participant, we segmented the entire LGN based on an anatomical atlas and additionally localized it functionally (LGN localizer^18^) (Fig. 1A). Within the LGN, we mapped the M- and P-LGN (M/P mapping^18^) (Fig. 1B) to test for functional differences in the M- and P-LGN between control and DD participants. In a further fMRI experiment, we assessed visual motion processing (motion experiment, Fig. 1C). This experiment was originally developed in the context of a different research question as a V5/MT localizer and here served as a quality control for the identified M- and P-LGN derived from the M/P mapping experiment.

### Overall LGN responses similar between control and developmental dyslexia participants

The LGN localizer (Fig. 1A) allowed us to functionally localize the entire LGN in each participant and assess whether DD participants may already differ from control participants in their overall LGN responses to visual stimulation. Such a difference between groups would indicate a general LGN deficit in DD that is not confined to any particular LGN subdivision. A mixed-design ANOVA of participants’ functional LGN responses with the between-subject factors of group (controls/DD) and sex (female/male) and the within-subject factors of hemisphere (left/right) and stimulation site (left/right visual hemifield) provided no support for such a global LGN deficit in DD. There were no significant main effects of group [*F*(1,50) = 0.223, *P* = 0.639, $\eta_{p}^{2}\;$ = 0.004] or sex [*F*(1,50) = 2.846, *P* = 0.098, $\eta_{p}^{2}\;$ = 0.054], nor any significant interaction involving both factors (all *P*'s ≥ 0.252, all $\eta_{p}^{2}\;$ ≤ 0.026), suggesting that overall LGN responses to the visual checkerboard stimulation were similar in control and DD participants.

### Altered M-LGN response in participants with developmental dyslexia

**Figure 3:**
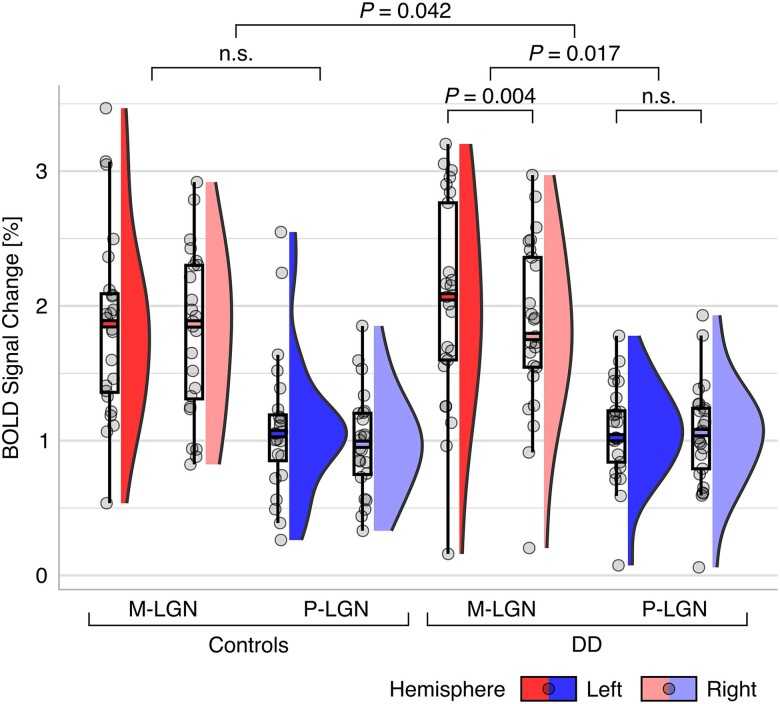
**Bilateral M/P-LGN BOLD responses in control (*n* = 24) and developmental dyslexia (*n* = 25) participants.** The figure displays box plots overlaid with individual data-points alongside colour-coded density plots. M-LGN responses are coded in red. P-LGN responses are coded in blue. Left and right-hemispheric responses are coded in saturated and light colours, respectively. Responses are averaged across stimulus-type to reveal the significant Group × Subdivision × Hemisphere interaction. BOLD = blood oxygen level-dependent; DD = developmental dyslexia; LGN = lateral geniculate nucleus; M = magnocellular; n.s. = not significant; P = parvocellular.

We then tested our first hypothesis that DD is associated with specific alterations of the M-LGN. For this purpose, we functionally defined each participant's M- and P-subdivision (Fig. 2A and B). We computed a mixed-design ANOVA of participants’ subdivision-specific LGN responses with the between-subject factors of group (controls/DD) and sex (female/male) and the within-subject factors of subdivision (M-LGN/P-LGN), stimulus-type (M-stimulus/P-stimulus), and hemisphere (left/right). The analysis revealed significant main effects of the factors subdivision [M-LGN > P-LGN responses; *F*(1,45) = 230.190, *P* = 2.6 × 10^−19^, $\eta_{p}^{2}\;$ = 0.836], stimulus-type [M-stimulus > P-stimulus responses; *F*(1,45) = 222.001, *P* = 5.2 × 10^−19^, $\eta_{p}^{2}\;$ = 0.831] and hemisphere [left > right responses; *F*(1,45) = 7.252, *P* = 0.01, $\eta_{p}^{2}\;$ = 0.139], while the main effects of the factors group [*F*(1,45) = 0.027, *P* = 0.871, $\eta_{p}^{2}\;$ = 0.001] and sex [*F*(1,45) = 0.703, *P* = 0.406, $\eta_{p}^{2}\;$ = 0.015] were non-significant. Importantly, there was a significant three-way interaction of Group × Subdivision × Hemisphere [*F*(1,45) = 4.361, *P* = 0.042, $\eta_{p}^{2}\;$ = 0.088; Fig. 3]. Conversely, the same interaction including the stimulus-type factor was not significant [*F*(1,45) = 0.737, *P* = 0.395, $\eta_{p}^{2}\;$ = 0.016], likely reflecting the partial functional overlap (i.e. rather than strict dichotomy) in the response profiles of M and P neurons.^27-29^ The observed three-way interaction suggested a difference in the Subdivision × Hemisphere interaction between the two groups of control and DD participants. Given previous results of potential left lateralization of sensory thalamus alterations in DD,^9,10,19^ we expected a significant Subdivision × Hemisphere interaction in the DD but not in the control group. In line with this expectation, two subsequent within-group repeated-measures ANOVAs revealed a significant interaction of Subdivision × Hemisphere in DD participants [*F*(1,24) = 6.531, *P* = 0.017, $\eta_{p}^{2}\;$ = 0.214, at Bonferroni-adjusted significance level *α* = 0.025], while this interaction was non-significant in controls [*F*(1,23) = 0.724, *P* = 0.403, $\eta_{p}^{2}\;$ = 0.031] (Fig. 3). *Post hoc* paired *t*-tests showed that within the DD group, the observed Subdivision × Hemisphere interaction was driven by significant hemispheric differences in functional responses between the left and right M-LGN [*t*(24) = 3.199, *P* = 0.004, *d* = 0.64, two-tailed at Bonferroni-adjusted significance level *α* = 0.025]. M-LGN responses in DD participants were significantly stronger in the left than right hemisphere. In contrast, there were no differences in P-LGN responses between hemispheres [*t*(24) = 0.520, *P* = 0.608, *d* = 0.104] (Fig. 3). Additional exploratory *post hoc* independent *t*-tests, prompted by a reviewer, revealed no further significant differences between groups. Overall, these results suggest that, unlike typical readers, DD participants exhibit functional response alterations, which are characterized by a notable increase in hemispheric asymmetry specifically affecting the M-LGN. Consistent with post-mortem human studies,^4^ these findings provide first evidence that DD is associated with alterations of the M-LGN also *in vivo*.

**Figure 4:**
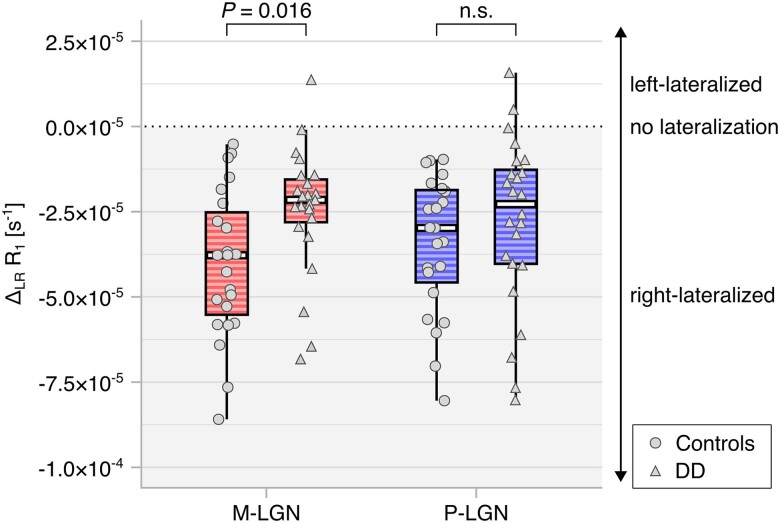
**Myelination of the M-LGN (*n* = 23 controls, *n* = 23 developmental dyslexia) and P-LGN (*n* = 23 controls, *n* = 24 developmental dyslexia).** Laterality in myelination was assessed using difference scores between the R_1_ values of the M/P-LGN subdivisions in the left and right hemispheres (i.e. Δ_LR_ R_1_). The sign of delta indicates the direction of lateralization (i.e. negative Δ = right-lateralized, positive Δ = left-lateralized). The dotted line indicates equal contributions of R_1_ from the left and right M/P-LGN to the difference score (i.e. no lateralization in myelination). The figure displays box plots overlaid with individual data-points. Data from control participants are represented by light grey circles, while data from participants with developmental dyslexia (DD) are represented by light grey triangles. Independent non-parametric Mann-Whitney *U*-tests revealed significant group differences in the lateralization of R_1_ for the M-LGN (*U* = 155.0, *P* = 0.016, two-tailed at Bonferroni-adjusted significance level *α* = 0.025) but not P-LGN (*U* = 218.0, *P* = 0.217). LGN = lateral geniculate nucleus; M = magnocellular; n.s. = not significant; P = parvocellular.

In an additional exploratory analysis of the quantitative T_1_ data (Supplementary material, ‘Methods’ section), we found that the altered functional lateralization of the M-LGN in DD may relate to differences in the underlying myelination of this subdivision between controls and individuals with DD (Fig. 4). While both the M- and P-LGN featured a rightward lateralization pattern in myelination in the two groups, the myelin content of the M-LGN was significantly shifted towards the left hemisphere in DD (*U* = 155.0, *P* = 0.016, two-tailed at Bonferroni-adjusted significance level *α* = 0.025) as compared to control participants (i.e. a tendency towards greater leftward lateralization in DD). Analogous to the fMRI results, the lateralization of the P-LGN did not differ between groups (*U* = 218.0, *P* = 0.217; Fig. 4).

### No differences in M/P-LGN localization accuracy between groups

First, there were no significant differences in the size of individually defined entire LGN masks (outlined in black in Fig. 2) between control and DD participants, either for the left LGN [128.2 ± 17.4 mm^3^ in controls versus 124.5 ± 14.0 mm^3^ in DD; *t*(52) = 0.838, *P* = 0.406, *d* = 0.228, two-tailed] or right LGN [136.1 ± 17.0 mm^3^ in controls versus 132.4 ± 17.7 mm^3^ in DD; *t*(52) = 0.793, *P* = 0.432, *d* = 0.216, two-tailed]. Second, the identified M/P-LGN maps were evaluated against the anatomically informed criterion that, for each participant, the M-LGN should be consistently located more medially than the P-LGN^12,18^ (Fig. 2B) (Supplementary material, ‘Methods’ section). Participants who did not meet this criterion (*n* = 4 controls, *n* = 1 DD) were excluded from the M/P-LGN analyses. There were also no differences in the size of the identified M/P-LGN maps between groups in either hemisphere in the final sample (all *P*'s ≥ 0.512, all *d's* ≤ 0.189). Finally, there were no significant differences between groups in behavioural performance on the contrast decrement detection task during the M/P mapping experiment (all *P*'s > 0.4; Supplementary material, ‘Results’ section).

### M/P-LGN maps show expected functional response properties

Next, we verified that the identified M/P-LGN subdivision maps showed the expected functional response properties of the M-LGN and P-LGN (Supplementary material, ‘Methods’ section). To this end, we analysed the subdivision-specific LGN responses in the independent visual motion experiment. Based on the known response properties of M-LGN and P-LGN neurons, BOLD responses to visual motion should be stronger in the identified M- than P-LGN.^6^ As expected, a mixed-design ANOVA of participants’ functional M/P-LGN responses to the contrast motion versus static with the between-subject factor of group (controls/DD) and the within-subject factors of subdivision (M-LGN/P-LGN) and hemisphere (left/right) revealed a significant main effect of the factor subdivision with stronger BOLD responses in the identified M- than P-LGN across participants [*F*(1,46) = 57.621, *P* = 1.194 × 10^−9^, $\eta_{p}^{2}\;$ = 0.556]. There was no main effect [*F*(1,46) = 0.181, *P* = 0.673, $\eta_{p}^{2}\;$ = 0.004] nor any interaction (all *P*'s ≥ 0.268, all $\eta_{p}^{2}\;$ ≤ 0.027) with the factor group, suggesting that the identified subdivisions in both groups adhered to the expected functional response properties.

### M-LGN response predicts key deficit in males with developmental dyslexia

**Figure 5:**
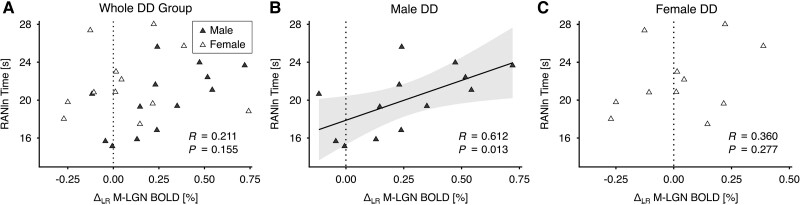
**M-LGN response in developmental dyslexia participants (*n* = 25), and its behavioural relevance for rapid automatized naming of letters and numbers**. (**A**) M-LGN response, quantified as a difference score between the blood oxygen level-dependent (BOLD) responses of the left and right M-LGN (i.e. Δ_LR_ M-LGN BOLD) in male (*n* = 13, dark triangles) and female participants (*n* = 12, bright triangles) with developmental dyslexia (DD). The dotted line indicates equal functional contributions of the left and right M-LGN to the difference score (i.e. no functional lateralization). There was no correlation between M-LGN difference scores (in %) and rapid automatized naming of letters and numbers (RANln; in s) across the whole DD group. (**B** and **C**) M-LGN difference scores correlated positively with the reaction time on RANln in (**B**) male DD (*n* = 13), but not in (**C**) female DD participants (*n* = 11). The plot in **B** shows the least squares correlation fit, including the 95% confidence interval (light grey shaded area) for the correlation coefficient *R*, in male participants with DD. LGN = lateral geniculate nucleus; M = magnocellular.

In human DD research, a commonly used diagnostic task is rapid automatized naming (RAN). In this task, participants name a series of visually presented familiar items (e.g. letters and numbers) aloud as quickly and accurately as possible.^30^ RAN ability is an important predictor of reading fluency and poses a key deficit in DD across the lifespan.^20^ Importantly, slow reaction times on RAN for letters and numbers (RANln) have previously been linked to functional and structural alterations of the sensory thalami and their connections to the cerebral cortex in DD.^10,19^ We therefore expected that the reaction times on RANln would be associated with M-LGN alterations in DD. To test this hypothesis, we correlated M-LGN alterations, quantified as a difference score between the BOLD responses of the left and right M-LGN (Δ_LR_ M-LGN BOLD), with the reaction times on RANln using one-tailed Pearson's correlation. To our surprise, there was no correlation between M-LGN difference scores and RANln performance across the whole DD group (*R* = 0.211, *P* = 0.155) (Fig. 5A). We realized that previous studies on the association between RANln and thalamo-cortical alterations in DD relied predominantly on male samples, limiting their predictive power for similar associations in female DD. We therefore tested the correlation separately within male and female DD participants (with Bonferroni correction for three statistical tests). The analysis revealed a significant correlation between M-LGN difference scores and RANln performance only in male DD participants (*R* = 0.612, *P* = 0.013, at Bonferroni-adjusted significance level *α* = 0.0167) (Fig. 5B). In contrast, the correlation within female DD participants (*R* = 0.36, *P* = 0.277) was not significant (Fig. 5C and Supplementary material, ‘Methods’ section).

## Discussion

Recent developments in high-field MRI have enabled the study of small brain structures, such as the subdivisions of human thalamic nuclei *in vivo*. Here we used this technical advance to image the human LGN and its M and P subdivisions in a large sample of adults with DD and matched control participants. Consistent with human post-mortem reports dating back to the 1990s,^4,5^ we found that individuals with DD show functional and structural response alterations specifically in the M-LGN. Our findings resolve the longstanding question of whether M-LGN alterations are also present in DD *in vivo* and give first indications about their potential sex-specific behavioural relevance.

Our finding of a different lateralization of the M-LGN in DD compared to controls is compatible with previous findings of left-lateralized sensory thalamic alterations in human DD.^5,9,19^ In the auditory pathway, histological changes occurred specifically in the left MGB in post-mortem brains of dyslexics.^5^*In vivo* MRI studies on DD have shown functional response changes and altered connectivity of the MGB, which were restricted to the left hemisphere.^9,19^ Previous findings on potential laterality of thalamic alterations in the visual processing pathway are less conclusive^4,10^: histopathological changes were found in the M but not P layers of the LGN; however, it is unclear which hemisphere(s) were affected.^4^ In addition, there is reduced structural connectivity between the left LGN and visual motion area V5/MT in DD, while connectivity results in the right hemisphere remain unclear.^10^ Recent behavioural findings point towards an altered lateralization in DD also in visual processing: typically reading individuals have a right hemifield advantage in detecting moving low-spatial frequency events, but this is not the case in DD.^31^ This is in line with aberrant left-hemispheric LGN-V5/MT connectivity^10^ and left-lateralized alterations in DD in the auditory sensory thalamus *in vivo* and post-mortem.^5,9,19^ The results of the present study do not permit us to adjudicate whether the divergent lateralization of the M-LGN is due to response differences within the left or right M-LGN. Within a predictive coding framework of brain processing, higher left-hemispheric LGN responses in DD could indicate larger levels of prediction error attributable to deficient left-lateralized cortico-thalamic feedback connections, resulting in poorer rapid naming performance.^10^ This speculation is, however, at odds with suggestions based on findings in the auditory thalamus in DD, where lower responses were found for speech tasks compared to other tasks.^19^ The exact nature of altered responses in DD in the sensory thalami therefore remains elusive.

Prior research in rat models of DD has shown that inducing microgyria in the developing neocortex leads to behavioural deficits and cellular changes in the sensory thalamus, akin to those observed in human DD post-mortem.^4,5,7^ Notably, these effects specifically manifest in male animals, while females are spared despite a similar cerebral cortex pathology.^32^ Our results in human adults partly parallel these findings, demonstrating a significant correlation between M-LGN alterations and behavioural deficits on RANln only in male individuals with DD. Impaired RANln performance has been repeatedly associated with left-hemispheric sensory thalamic alterations in male DD in previous human studies.^10,19^ The correlation between RANln and sensory thalamus alterations may thus be a hallmark of DD that is predominant in male individuals. However, we also note that our correlation analysis in male DD was based on a relatively modest subsample (*n* = 13) and needs replication in future studies. Finally, the absence of a significant interaction effect involving sex in our analysis leaves the question of sex dependency open. One possibility is that, unlike the observations in rat models, thalamic alterations in human DD may not be sex-dependent. Alternatively, the modest subsample sizes of males and females in this study may have limited our ability to detect potential sex-specific differences in M-LGN alterations (*cf*. M-LGN response between male and female DD in Fig. 5B and C).

Cross-sectional human case-control designs, as used in the present study, are confronted with the difficulty of discerning cause from secondary effects of the reading difficulties. This can be achieved, for example, through training or longitudinal studies, as has already been done for investigating the contribution of dorsal stream dysfunction to the development of DD.^33-36^ We cannot derive from our results how M-LGN alterations contribute to core DD symptoms. We have previously proposed two potential explanations.^10^ First, proficient reading and RANln performance require rapid, directed attentional shifts towards spatially successive letters and numbers. This visual-spatial skill is associated with a right-lateralized fronto-parietal attention network, including the ventral portion of the superior longitudinal fasciculus (SLF-III).^37,38^ Neurons of the M-LGN relay visual information to area V5/MT, which serves as a main input structure to the fronto-parietal attention network.^39^ Left-lateralized input from the M-LGN and a reduced right lateralization of M-LGN myelination, as observed in the current study, may perturb the dynamics of this right-lateralized attention network. This interference could explain diminished RANln performance and previous observations of lateralized behavioural deficits in children and adults with DD.^40-42^ Our second suggestion was that deficient RANln performance might result from a deficient top-down modulation of the LGN in response to fast-varying, predictable speech stimuli, i.e. visual articulatory movements.^10^ M-LGN neurons are known to process high temporal frequency visual information.^6^ Interestingly, DD is associated with a reduced structural connectivity between the LGN and area V5/MT in the left hemisphere.^10^ An imbalanced top-down modulation of M-LGN neurons could therefore contribute to a deficit in processing fast visual speech features in DD, which might be important for acquiring phonological skills during ontogeny.

In summary, we present a comprehensive 7 T MRI study involving a sample of *n* = 28 controls and *n* = 26 individuals with DD. We employed an extensive acquisition protocol at high spatial resolution to investigate the two main subdivisions of the human LGN in DD *in vivo*. Our results highlight M-LGN alterations as a key feature of DD. The findings confirm a longstanding hypothesis^4^ that DD is associated with alterations in the magnocellular system at the level of the LGN, but not the parvocellular system. In addition, the results suggest that M-LGN alterations are associated with reading-related behavioural scores in male DD. This emphasizes the need for a more in-depth understanding of sex-specific behavioural effects of sensory thalamic alterations in DD. This may be particularly helpful for the development of prospective treatment strategies, as targeting the thalamo-cortical system for example with complementary neurostimulation might be particularly effective in male individuals with DD.^43,44^

## Supplementary Material

awae235_Supplementary_Data

## Data Availability

The scripts used to generate the LGN hemifield and M/P-stimuli are publicly available.^18^ The motion experiment and fMRI analysis scripts have been made publicly available on the Open Science Framework (OSF; https://osf.io/bge75/).^22^ Raw MRI data cannot be made available as sharing these personal data is not covered by the ethics clearance. Single-subject data in native and MNI space (i.e. individual LGN, M-LGN, P-LGN and beta M-P maps) are available on OSF.
