# Supplementary material for: Dysfunction of the magnocellular subdivision of the visual thalamus in developmental dyslexia
Source: Brain. 2024 Aug 7;148(1):252–61. doi: 10.1093/brain/awae235 (PMC11706283; doi:10.1093/brain/awae235)
Supplement: awae235_Supplementary_Data [file awae235_supplementary_data.pdf]

Supplementary material

Dysfunction of the magnocellular subdivision of the visual thalamus in developmental dyslexia

Brain, awae235, https://doi.org/10.1093/brain/awae235

Methods

Participants

All participants were tested on literacy skills, including reading speed and comprehension[1] (LGVT) and spelling[2] (RT), as well as on rapid automatized naming of letters and numbers[3] (RANln), and word and non-word reading.[4] Participants' handedness was assessed with a German-adapted version of the abbreviated (10-item) Edinburgh Handedness Inventory[5] (Supplementary Table 1). Participants provided written informed consent in accordance with the Declaration of Helsinki before study participation. The study was approved by the ethics committee of the Medical Faculty, University of Leipzig, Germany. All participants were recruited and scheduled for data collection in the period from January 2017 to July 2019.

Since this is the first study to investigate M-LGN alterations in DD *in vivo*, there was no prior effect size available for a formal estimation of the required sample size. We sought to maximize the number of participants in both groups, considering the stringent safety criteria for high-field 7 T MRI (refer to the 'General participant inclusion criteria' section below) and the allocated MR data acquisition time.

Inclusion criteria for DD participants

Participants with DD were required to meet the following criteria to be included in the study: (i) reading accuracy and/or speed, as assessed by measures commonly used for diagnosis of DD in Germany (i.e. LGVT or non-word reading), of at least 1.5 standard deviations (SD) below the mean of the matched control group; and (ii) a life-long history of DD in the anamnesis. Participants with DD were recruited nationwide through print and online study advertisements.

General participant inclusion criteria

All participants had to fulfill the following inclusion criteria: (i) no prior history of neurological and/or psychiatric disorders, (ii) free of psychostimulant medication, (iii) no co-existing neurodevelopmental disorders other than dyslexia (e.g. dyscalculia, autism spectrum disorder), (iv) no hearing disabilities, (v) normal or corrected-to-normal visual acuity and (vi) a non-verbal IQ ≥ 85.

The first four criteria were assessed based on participants' self-reports and screening questionnaires including the Autism-Spectrum Quotient (AQ)[6] and a brief, self-designed 10-item questionnaire on the main symptoms of dyscalculia.[7,8] Visual acuity was assessed through the Freiburg Visual Acuity Test (FrACT3; https://michaelbach.de/fract/)[9,10] with a cutoff of +0.1 binocular logMAR to ensure normal visual acuity.[11] Non-verbal IQ was assessed with the German adaptation of the Wechsler Adult Intelligence Scale-Revised (HAWIE-R).[12]

Finally, all participants had to meet the local safety requirements for high-field MRI: no metal implants, free of tattoos and non-removable ferromagnetic jewellery, no dental amalgam restorations, complete medical documentation of all potentially relevant previous surgical procedures and accidents as well as no pregnancy in female participants (with the option to perform a rapid pregnancy test on site).

Display and visual stimulation software

Within the MRI system, participants viewed the screen from a total viewing distance of 35 cm, which subtended approximately 18 × 16 degrees of visual angle. Stimuli were generated on Linux using the Psychtoolbox,[13,14] implemented in GNU Octave, version 4.2.0,[15] and presented at a refresh rate of 60 Hz.

Physiological data recordings

We recorded participants' cardio-respiratory data throughout each fMRI experiment using an MRI-compatible Biopac System (Biopac Systems Inc., Goleta, CA, USA). Cardiac signals were recorded through a pulse oximeter placed on participants' left (non-dominant) index finger with a sampling rate of 100 Hz. Respiratory data were recorded through thoracic movements using a non-electrical pressure pad placed on participants' chest in combination with a respiration transducer. MR trigger pulses were recorded to synchronize physiological parameters to each MR volume.

High resolution 7 T functional MRI acquisition

High resolution functional MRI data were acquired using a gradient-echo echo-planar imaging (EPI) sequence with the following imaging parameters: 1.25 mm isotropic resolution in-plane, 1.20 mm slice thickness (no gap), TE = 16 ms, TR = 2000 ms, α = 80°, FoV = 192.5 × 192.5 × 48 mm^3, echo spacing = 0.78 ms, readout bandwidth = 1476 Hz/Px, GRAPPA = 3 and Partial Fourier (PF) of 6/8 in phase-encoding direction. Functional volumes (LGN localizer: single run of 136 volumes; M/P mapping experiment: four runs of 144 volumes each; motion experiment: single run of 130 volumes) were acquired with partial brain coverage (40 transverse slices). The number of slices and/or flip angle were adjusted in some participants (n = 4 controls and n = 5 DD) due to restrictions in energy absorption (i.e. specific absorption rate) typically associated with high-field MRI (minimum number of slices = 33, minimum flip angle = 69°). The reduction of the flip angle in these participants was well within the normal range of typical flip angle variation throughout the brain at 7 T.[16] In addition, we acquired one whole-brain EPI image with matching parameters to facilitate registrations between the functional and structural MRI data. To correct images for geometric distortions induced by magnetic field inhomogeneity, in each MRI session we acquired two gradient-echo datasets (Δ_{TE} = 1.02 ms) from which session-specific B_0 field-maps (voxel displacement) were computed.

High resolution 7 T structural MRI acquisition

High resolution whole-brain structural MRI data, including a conventional T_1-weighted image and a quantitative T_1 map, were obtained using a 3D-MP2RAGE sequence[17] with the following imaging parameters: 700 µm isotropic resolution, TE = 2.45 ms, TR = 5000 ms, TI_1/TI_2 = 900/2750 ms, α_1/α_2 = 5/3°, FoV = 224 × 224 × 168 mm^3, echo spacing = 6.8 ms, readout bandwidth = 250 Hz/Px, GRAPPA = 2 and 6/8 PF in phase-encoding direction. The acquisition took 10:57 minutes.

Preprocessing of fMRI data

Individual volumes of each run of the M/P mapping experiment and motion experiment were realigned to the first volume of the LGN localizer and unwarped based on the session-specific field-maps to correct for motion artefacts and EPI distortions. The whole-brain EPI image was also co-registered to this volume and subsequently used as the reference image for registering

the structural to the functional data. Unwarped functional data in native space were smoothed using a Gaussian filter with a full width at half maximum (FWHM) matching the voxel size (i.e. 1.25 × 1.25 × 1.2 mm). The time series of each voxel were high-pass filtered (1/128 Hz cutoff) to remove low-frequency noise and signal drift. The resulting images were used for the definition of M/P-LGN subdivisions and all further analyses.

As part of the quality control analysis for the M/P-LGN subdivisions in native space, the unwarped functional data were also normalized to standard space (MNI, Montreal Neurological Institute). To this end, the anatomical image was segmented into six tissue probability maps (grey matter, white matter, cerebrospinal fluid, soft tissue, bone, image background). These tissue class maps were then nonlinearly registered to the 1 mm MNI brain template and derived registration parameters were applied to the functional data. The registered functional data were smoothed using a Gaussian filter with a FWHM matching the voxel size. Finally, the time series of each voxel were high-pass filtered at a 1/128 Hz cutoff.

Head motion

Head motion was assessed by computing the maximum translational and rotational displacements across each run of each fMRI experiment from the six motion parameters (three translation, three rotation) obtained from data realignment in SPM.[18] Maximum translational displacements (TD) corresponded to the maximum difference between total TDs calculated as the square root of the sum of squared x, y and z-direction displacements. Maximum rotational displacements (RD) corresponded to the maximum difference between total RDs calculated as the sum of the absolute RDs in the three directions. We also computed the mean framewise displacement (FD), which accounts for the mean translational and rotational head motion between adjacent slices. RDs were converted from degrees to millimetres assuming a spherical surface with a 50 mm radius. Independent *t*-tests comparing control and DD participants on these displacement measures (i.e. TD, RD and FD) for each fMRI experiment revealed no significant group differences (LGN localizer: all P's $\geq$ .10, M/P mapping experiment: all P's $\geq$ .16, motion experiment: all P's $\geq$ .07).

fMRI data analysis

For each fMRI experiment, the preprocessed data in native space were analysed using single-participant general linear models (GLM) for block designs.[19] For each participant and fMRI experiment, the two conditions of interest (i.e. LGN localizer: left hemifield checkerboard versus right hemifield checkerboard; M/P mapping experiment: M-stimulation versus P-stimulation; motion experiment: motion versus static) were modeled as box-car functions convolved with the canonical hemodynamic response function. For the M/P mapping experiment, data from the four runs were concatenated into a single session and additional regressors were added to account for between-run variance.[18] Motion parameters (three translation and three rotation) derived from realignment and 16 physiological parameters obtained from the PhysIO toolbox[20] were also modeled as regressors of no interest to account for motion and cardio-respiratory-related variance. The physiological regressors included models of cardiac (six regressors) and respiratory phases (eight regressors) computed using Fourier expansions of different order, based on RETROICOR.[21] Physiological regressors also included models of heart rate variability[22] and respiratory volume per time.[23] Including models of physiological noise and motion parameters as nuisance regressors has been shown to substantially increase the signal-to-noise ratio in the LGN at 7 T.[24] Due to technical problems, physiological parameters of six participants could not be acquired and were not considered in the respective design matrices.

LGN definition

To segment the LGN in each participant and to demarcate it from adjacent visual brain structures, we leveraged a publicly available high resolution 7T probabilistic LGN atlas.[25] This atlas is available in high resolution 0.4 mm template space as well as in MNI 1 mm standard space.[26] To map the bilateral LGNs in each participant's native space, we performed the following steps: We used the *antsRegistration* command, implemented in the Advanced Normalization Tools (ANTs) software package,[27] to register the high resolution 0.4 mm template to each participant's quantitative T_1 image. The registrations were run with rigid and affine linear registrations in combination with nonlinear symmetric normalization (SyN). All registrations were visually inspected for potential misalignments. In some participants, the local vessel architecture around the LGN affected the quality of the registrations and required the use of additional landmark information (i.e. medial-lateral and inferior-superior LGN extent in two central LGN slices) in the linear registration initialization step. After quality control, the

obtained participant-specific registration parameters were applied to the left and right probabilistic LGN atlas. Following registrations of these masks to the functional image data, the registered probabilistic LGN masks were set to a threshold of 35% overlap to confine final entire LGN mask sizes to anatomically plausible volumes (left LGN: 128.2 ± 17.4 mm^3 in controls versus 124.5 ± 14.0 mm^3 in DD; right LGN: 136.1 ± 17.0 mm^3 in controls versus 132.4 ± 17.7 mm^3 in DD).[28] In addition, we also verified that the structurally defined entire LGN masks coincided with the functional LGN activations derived from the LGN localizer experiment. This was the case in all participants. The participant-specific entire LGN masks were used to spatially constrain the individual beta M-P maps to define M and P subdivisions by applying a 20/80% volume threshold.

Quality control of M/P-LGN mapping

We performed two main quality control analyses to assess the localization accuracy of the identified M/P-LGN subdivision maps:

(1) To assess the structural plausibility of the individually defined M/P-LGN maps, we first computed beta M-P maps for each participant in MNI standard space. These maps were then masked with an openly available LGN probabilistic atlas (at a threshold of 35% overlap) in MNI 1 mm standard space,[26] and M- and P-LGN were computed by applying a 20/80% volume threshold. For each participant, we calculated the centres of mass of the identified M- and P-LGN subdivisions as a proportion of individual LGN extent. Based on prior anatomical knowledge, we expected the M-LGN to be located more medially than the P-LGN.[18,25] This was the case for the majority of participants and those who did not meet this criterion (n = 4 controls, n = 1 DD) were excluded from all further analyses. We performed this control analysis in MNI standard space to account for potential differences in LGN orientation between participants (due to differences in MRI slice angulation) in native space.

(2) We also assessed the functional plausibility of the identified M- and P-LGN subdivision maps by examining their responses to visual motion from the motion experiment. Based on the known response properties of M and P neurons, functional responses to visual motion were expected to be stronger in the identified M- than P-LGN.[29]

Structural brain data analysis

In an additional analysis, we explored potential group differences in the underlying microstructure of the individually defined M- and P-LGN subdivision maps based on the quantitative T_1 maps of the MP2RAGE data. We first co-registered each participant's T_1 image with the functional data. We then extracted the inverse of the longitudinal relaxation time, R_1, from the functionally defined M- and P-LGN maps as an indicator of the underlying tissue myelination of both subdivisions. In line with the results of the fMRI analysis, we aimed to explore group differences in the lateralization of the M/P-LGN subdivisions. We therefore computed difference scores between the extracted R_1 values from these maps in the left and right hemispheres (i.e. Δ_{LR} R_1) to assess potential differences in the laterality of myelination. As the analysis heavily rests on the accuracy of the identified M- and P-LGN subdivisions, only the R_1 data of participants whose functionally defined subdivision maps met the quality control criteria were considered (i.e. $n = 24$ controls, $n = 25$ DD).

Results

Behavioural performance

Behavioural performance (% of correct responses) during the M/P mapping experiment (report of contrast decrements within each block) was analysed using a mixed-design ANOVA with group (controls/DD) as between-subject factor and stimulus-type (M-stimulus/P-stimulus) as within-subject factor. There was no significant main effect of group ($F(1, 41) = 0.614$, $P = 0.438$, $\eta_p^2 = 0.015$), no main effect of stimulus-type ($F(1, 41) = 0.420$, $P = 0.520$, $\eta_p^2 = 0.010$), nor an interaction between both factors ($F(1, 41) = 0.006$, $P = 0.937$, $\eta_p^2 = 0.0001$). Mean performance across groups and stimuli was 43.91 ± 21.56%. This relatively low performance could be due to errors in the use of the response keys: participants frequently reported that they found the response keys counterintuitive, tending toward not pressing any key when they detected 0 targets and using their index, middle and ring fingers to report 1, 2 and 3 targets, respectively, thereby shifting the response keys. Such a strategy could explain the low overall accuracy. The original study that introduced the M/P-mapping experiment reported an accuracy of 71-75%, which we assume is due to conducting the experiment multiple times on the same participants, including two of the study authors.[18]

Supplementary Table 1 Demographic data and diagnostic test performance in controls and developmental dyslexia

	Control participants (*n* = 28)	DD participants (*n* = 26)	Δ (Controls, DD)
**Demographic data**			
Age, mean ± SD [years]	26.5 ± 6.2	27.8 ± 7.4	$U = 331.5, P = 0.579$
Sex [females | males]	15 | 13	13 | 13	-
Handedness [right | left]	28 | 0	26 | 0	-
Education [12 | 10 years]	28 | 0	21 | 5	-
**Diagnostic tests, mean ± SD**			
Non-verbal intelligence[a]	120.4 ± 9.70	117.3 ± 12.9	$t(52) = 1.0, P = 0.323$
Spelling[b]	107.8 ± 10.0	83.5 ± 9.9	$U = 682.5, P = 3.580\times10^{-8}$
Reading speed[c]	59.0 ± 9.2	42.5 ± 7.0	$U = 679.5, P = 4.823\times10^{-8}$
Reading comprehension[c]	63.2 ± 9.6	46.9 ± 8.9	$t(52) = 6.5, P = 3.576\times10^{-8}$
RANln[d]			
Time [s]	16.8 ± 2.2	21.0 ± 3.5	$t(41.1) = -5.3, P = 4.402\times10^{-6}$
Errors [%]	0.3 ± 0.8	1.2 ± 2.3	$U = 264.0, P = 0.029$
Word reading[e]			
Time [s]	34.2 ± 6.3	52.5 ± 15.4	$t(32.7) = -5.6, P = 3.002\times10^{-6}$
Errors [%]	0.7 ± 1.3	4.4 ± 4.3	$U = 153.5, P = 8.057\times10^{-5}$
Non-word reading[e]			
Time [s]	68.5 ± 12.4	134.1 ± 46.10	$U = 46.0, P = 3.871\times10^{-8}$
Errors [%]	6.5 ± 4.6	26.8 ± 13.2	$U = 39.5, P = 1.858\times10^{-8}$

Δ (Controls, DD), statistical test of group difference; independent *t*-test or Mann-Whitney *U*-test, where appropriate.

[a] HAWIE-R, standard scores (mean = 100, SD = 15).[12]

[b] German spelling test (RT), standard scores (mean = 100, SD = 10).[2]

[c] German reading speed and comprehension test (LGVT), *t*-standard scores (mean = 50, SD = 10).[1]

[d] RANln, composite score of rapid automatized naming of letters and numbers, raw scores.[3]

References

1. Schneider W, Schlagmüller M, Ennemoser M. *Lesegeschwindigkeits- Und -Verständnistest Für Die Klassen 6-12: LGVT 6-12*. Hogrefe; 2007.
2. Kersting M, Althoff K. *Rechtschreibungstest: RT*. Hogrefe; 2004.
3. Denckla MB, Rudel RG. Rapid 'automatized' naming (R.A.N.): Dyslexia differentiated from other learning disabilities. *Neuropsychologia*. 1976;14:471-479.
4. Schulte-Körne G. *Lese-Rechtschreibstörung Und Sprachwahrnehmung. Psychometrische Und Neurophysiologische Untersuchungen Zur Legasthenie.* Waxmann; 2001.
5. Oldfield RC. The assessment and analysis of handedness: the Edinburgh inventory. *Neuropsychologia*. 1971;9:97-113.
6. Baron-Cohen S, Wheelwright S, Skinner R, Martin J, Clubley E. The Autism-Spectrum Quotient (AQ): Evidence from Asperger syndrome/high-functioning autism, males and females, scientists and mathematicians. *J Autism Dev Disord*. 2001;31:5-17.
7. Butterworth B. Developmental Dyscalculia. In: Campbell JID, ed. *Handbook of Mathematical Cognition*. Psychology Press; 2005:455-468.
8. Shalev RS. Developmental Dyscalculia. *J Child Neurol*. 2004;19:765-771.
9. Bach M. The Freiburg Visual Acuity Test - automatic measurement of visual acuity. *Optometry and Vision Science*. 1996;73:49-53.
10. Bach M. The Freiburg Visual Acuity Test - Variability unchanged by post-hoc re-analysis. *Graefe's Archive for Clinical and Experimental Ophthalmology*. 2006;245:965-971.
11. Colenbrander A. Aspects of vision loss – visual functions and functional vision. *Vis Impair Res*. 2003;5:115-136.
12. Tewes U. *Hamburg-Wechsler-Intelligenztest Für Erwachsene (HAWIE-R)*. Revision 1991. Hans Huber Verlag; 1991.
13. Pelli DG. The VideoToolbox software for visual psychophysics: transforming numbers into movies. *Spat Vis*. 1997;10:437-442.
14. Brainard DH. The Psychophysics Toolbox. *Spat Vis*. 1997;10:433-436.

15. Eaton J, Bateman D, Hauberg S, Wehbring R. GNU Octave. A high-level interactive language for numerical computations. Edition 4 for Octave version 4.2.0. Published 2016. Accessed June 3, 2024. https://docs.octave.org/octave-4.2.0.pdf

16. Vaughan JT, Garwood M, Collins CM, et al. 7T vs. 4T: RF power, homogeneity, and signal-to-noise comparison in head images. *Magn Reson Med*. 2001;46:24-30.

17. Marques JP, Kober T, Krueger G, van der Zwaag W, van de Moortele PF, Gruetter R. MP2RAGE, a self bias-field corrected sequence for improved segmentation and T1-mapping at high field. *Neuroimage*. 2010;49:1271-1281.

18. Denison RN, Vu AT, Yacoub E, Feinberg DA, Silver MA. Functional mapping of the magnocellular and parvocellular subdivisions of human LGN. *Neuroimage*. 2014;102:358-369.

19. Friston KJ, Holmes AP, Worsley KJ, Poline JP, Frith CD, Frackowiak RSJ. Statistical parametric maps in functional imaging: A general linear approach. *Hum Brain Mapp*. 1994;2:189-210.

20. Kasper L, Bollmann S, Diaconescu AO, et al. The PhysIO toolbox for modeling physiological noise in fMRI data. *J Neurosci Methods*. 2017;276:56-72.

21. Glover GH, Li TQ, Ress D. Image-based method for retrospective correction of physiological motion effects in fMRI: RETROICOR. *Magn Reson Med*. 2000;44:162-167.

22. Chang C, Cunningham JP, Glover GH. Influence of heart rate on the BOLD signal: The cardiac response function. *Neuroimage*. 2009;44:857-869.

23. Birn RM, Smith MA, Jones TB, Bandettini PA. The respiration response function: The temporal dynamics of fMRI signal fluctuations related to changes in respiration. *Neuroimage*. 2008;40:644-654.

24. Hutton C, Josephs O, Stadler J, et al. The impact of physiological noise correction on fMRI at 7 T. *Neuroimage*. 2011;57:101-112.

25. Müller-Axt C, Eichner C, Rusch H, et al. Mapping the human lateral geniculate nucleus and its cytoarchitectonic subdivisions using quantitative MRI. *Neuroimage*. 2021;244:118559.

26. Müller-Axt C, Eichner C, Rusch H, et al. Data repository: Mapping the human lateral geniculate nucleus and its cytoarchitectonic subdivisions using quantitative MRI. doi:10.17605/OSF.IO/TQAYF

27. Avants BB, Epstein CL, Grossman M, Gee JC. Symmetric diffeomorphic image registration with cross-correlation: evaluating automated labeling of elderly and neurodegenerative brain. *Med Image Anal*. 2008;12:26-41.

28. Andrews TJ, Halpern SD, Purves D. Correlated size variations in human visual cortex, lateral geniculate nucleus, and optic tract. *Journal of Neuroscience*. 1997;17:2859-2868.

29. Nassi JJ, Callaway EM. Parallel processing strategies of the primate visual system. *Nat Rev Neurosci*. 2009;10:360-372.
